# Acousto-holographic investigation of the changes in morphology and cellular biomechanics under oxidative stress

**DOI:** 10.1038/s41598-025-32709-0

**Published:** 2026-02-09

**Authors:** Zeynep Karavelioglu, Rahmetullah Varol, Merve Sevgi, Gulsum Gencoglan, Gokhan Bora Esmer, Rabia Cakir, Yasemin Basbinar, Huseyin Uvet

**Affiliations:** 1 Department of Bioengineering, Yildiz Technical University, Istanbul, 34220 Turkey; 2 Department of Mechatronics Engineering, Yildiz Technical University, Istanbul, 34349 Turkey; 3 Oncology Institute, Dokuz Eylul University, Izmir, 35330 Turkey; 4 Department of Dermatology, Medicana Hospital, Istanbul, 34158 Turkey; 5 Department of Electrics and Electronics Engineering, Marmara University, Istanbul, 34722 Turkey; 6 Present Address: Department of Health Sciences and Technology, ETH Zürich, Zürich, 8092 Switzerland; 7 Present Address: Mechano-Genomics Group, Division of Biology and Chemistry, Paul Scherrer Institute, Villigen, 5232 Switzerland; 8 Present Address: Universität der Bundeswehr München, 85577 Munich, Germany

**Keywords:** Apoptosis, Cell stiffness, Diagnosis, Drug discovery, Holographic microscopy, Microscopy, Cell biology

## Abstract

Oxidative stress is an important cellular phenomenon that’s necessary for the cell to maintain its metabolic activities but causes adverse complications due to abnormal accumulation. It may cause some changes in the mechanical structure and behavior of the cells by affecting the cell membrane and cytoskeleton structure and triggering apoptosis. Moreover, it’s closely related to many conditions such as cancer, neurodegenerative diseases, and aging, making it important to examine and understand this phenomenon very well at the cellular level. This study reports a label-free and contact-free platform to image the morphological and mechanical behavior of live cells using an acousto-holographic microscope. F-actin fluorescence staining and Annexin V-FITC/PI staining were also performed to support our results. Different stages of apoptosis can be morphologically distinguished by our method while mapping the elasticity modulus distribution of the cells. It’s found that C2C12 myoblast cells, which initially had the highest elasticity modulus, have less resistance to apoptosis and undergo a more significant decrease in elasticity modulus under oxidative stress. HCT 116 cancer cells, which were the softest at the beginning, experience a weaker decrease in elasticity modulus under oxidative stress compared to C2C12 and HUVEC cells. This supports the resistance of HCT 116 cells against apoptosis.

## Introduction

 Oxidative stress occurs as a result of the abnormal accumulation of the reactive oxygen species (ROS) in the cells and imbalances in the production-detoxification process of the ROS^1^. ROS are associated with many conditions such as cancer, neurodegenerative diseases, cardiovascular diseases, and aging^2^. It is also known that ROS have an important effect on the signaling pathways that enable the programmed cell death, also known as apoptosis, which is necessary for multicellular organisms to develop and maintain their vital activities^3^.

Cells generate and sustain mechanical forces and respond to physical cues through processes such as reorganization of the cytoskeleton and force generation^4^. Furthermore, it is known that oxidative stress causes actin depolymerization by affecting the cytoskeleton structure and induces some changes in the mechanical behavior of the cells^5^. Stiffness is a crucial mechanical property of cells, and it is associated with several vital functions including migration, motility, and adhesion properties^6–8^. Moreover, cell stiffness can regulate cell growth via apoptosis and plays a crucial role in the cell’s fate^9,10^. In this context, examining the connection between the cell stiffness, oxidative stress, and apoptosis has great importance for biomedical studies to better understand the effect of the stress on a cell’s mechanical behavior. Cell stiffness is widely measured by Atomic Force Microscopy (AFM). Using the AFM technique, the elastic modulus of cells is quantitatively determined in a high-resolution way^7^. However, although AFM is considered a gold standard method for measuring cell stiffness, it has several disadvantages such as low scanning speed and thermal shift in images^11,12^. Also, its use in the clinic is limited due to time-consuming analysis time, difficult handling, and the possibility of damaging the cell, especially in live cell imaging.

Today, different methods based on different aspects such as biochemistry, molecular biology, immunology, and morphology are available to examine apoptosis^13^. In the context of molecular biology based methods, although DNA fragmentation by DNA gel electrophoresis is a user-friendly method, it is only semi-quantitative with low specificity and is not suitable to localize apoptotic cells. This method is only suitable for detecting large-scale apoptotic cells in the middle and late stages, not those in the early stages^14^. The in situ DNA nick end labeling (TUNEL) method, on the other hand, catalyzes the binding of labeled dUTPs such as fluorescein isothiocyanate (FITC) or biotin to 3’OH groups, based on DNA fragmentation during apoptosis, using terminal deoxynucleotidyl transferase (TdT). Even though it is a relatively more quantitative method, false positives may still occur^15^. RT-qPCR can also be used to demonstrate apoptosis-related gene expression. However, it cannot measure the related protein expression^16^.

Within the context of immunological methods, Enzyme-Linked Immunosorbent Assay (ELISA) is a highly sensitive method. However, over-lysis of cells may also cause staining of DNA fragments within the nucleus. Although this method is suitable for large-scale qualitative and quantitative analysis of apoptosis, it cannot be used to localize apoptotic cells^13^. The Annexin V-PI method allows the examination of apoptosis at early, middle, and late stages in a very sensitive manner. However, this method is quite expensive, and the flow cytometer must be used very precisely to avoid damaging the cell membrane while performing it^17^.

Considering the morphological approaches used to examine apoptosis, characteristic morphological features exhibited at different stages of apoptosis can be examined by histological staining such as hematoxylin and eosin (HE)^18^ or Giemsa^19^ staining. Additionally, apoptosis can also be evaluated by fluorescence microscopy using fluorescence dyes such as Hoechst^20^, 4’,6-diamidino-2-phenylindole (DAPI)^21^, and acridine orange (AO)^22^. Electron microscopes can also be used to determine the morphology of apoptotic cells. However, the staining requirement in these methods limits the ability of samples stained once to be reused for further studies. Also, the cost of the dyes makes these methods far from being budget-friendly. In addition, techniques such as confocal microscope and electron microscope require both long analysis times and an expert operator.

In this study, different stages of apoptosis occurring under oxidative stress caused by hydrogen peroxide (H_2_O_2_) were examined in a label-free and high-resolution manner with an acousto-holographic microscope designed to analyze the morphology and elasticity modulus of cells^23^. At the same time, the changing elasticity modulus distribution of the cell under oxidative stress was mapped. Conditions such as pyknosis and karyorrhexis, which the cell undergoes during the apoptosis process, were visualized without any chemical markers or probes. In addition, the changes in nucleus size under oxidative stress were calculated according to the changing cytoplasm size. Also, in order to support the acousto-holographic data, the changes in the actin skeleton were examined fluorescently, and the apoptosis process of the cells was evaluated by Annexin V-FITC/PI staining.

In summary, our study allows the morphological analysis of living cells in a real-time and label-free manner. Additionally, the morphological information can be correlated with real-time elasticity modulus maps. This study has the potential to make a valuable contribution to the simultaneous monitoring of the apoptosis process at the single-cell level and its effect on the biomechanical behavior of the cell. In addition, it may provide both a diagnostic-monitoring and drug discovery testing platform for various areas such as cancer and age-related diseases, where the apoptosis process is of critical importance.

## Results and discussion

### Acousto-holographic investigation of the cell mechanics

**Fig. 1:**
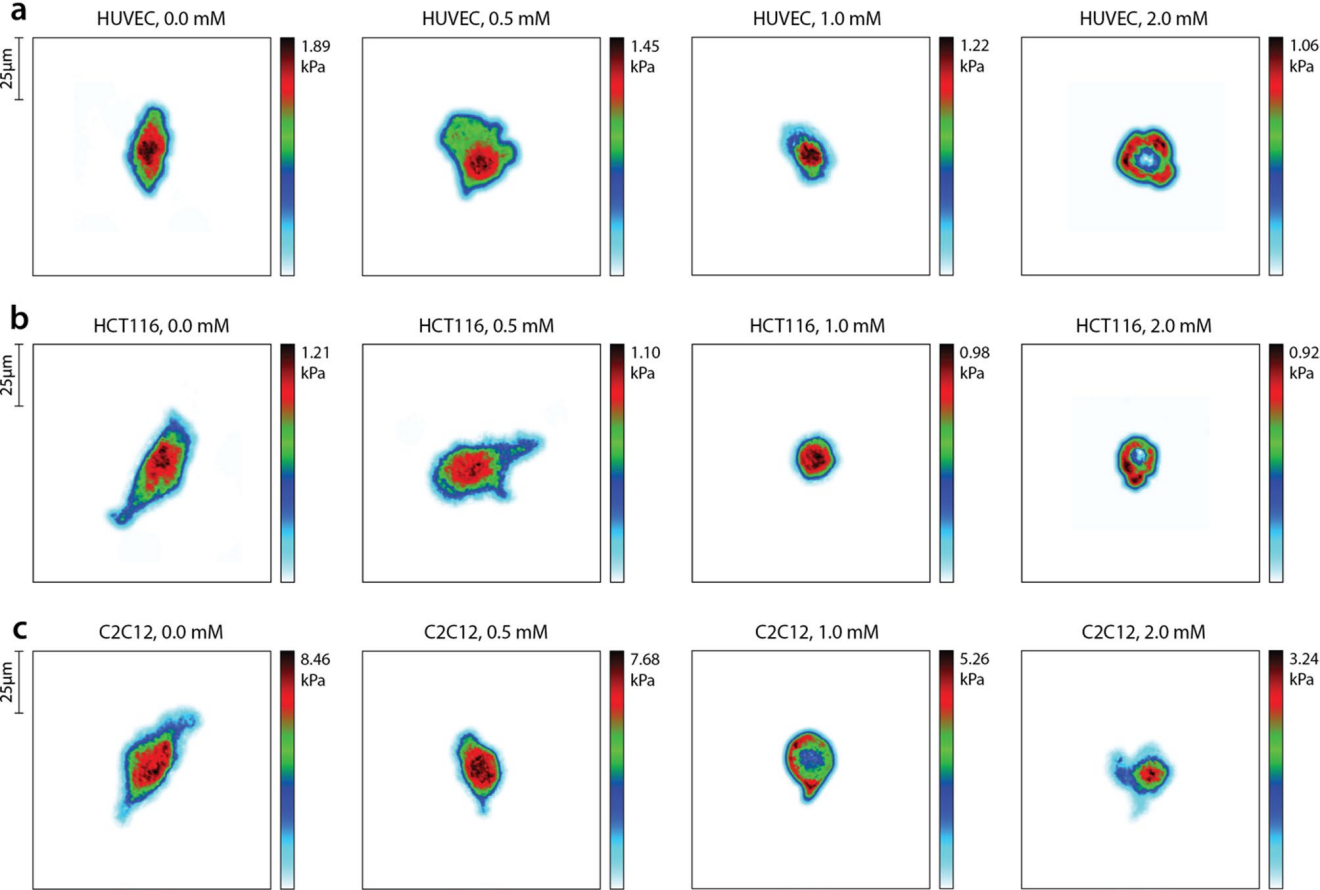
Elasticity modulus mapping of (**a**) HCT 116, (**b**) C2C12, and (**c**) HUVEC cells subjected to 0 mM, 0.5 mM, 1 mM, and 2 mM concentrations of H_2_O_2_. The elasticity modulus distribution was calculated by subjecting the cells to an acoustic pressure and measuring the deflection of each point via a holographic imaging system, as detailed in the Methods section.

Longitudinal elasticity modulus values obtained using the acousto-holographic technique are given in this section. These were calculated by measuring the displacement of the cell membrane in response to a periodic acoustic signal. The displacement was associated with elasticity modulus using the Hertzian contact model^24^ through the association of acoustic pressure with cellular membrane deflection. The indentation depth in our experiments is determined by the displacement of the cell membrane in response to the acoustic pressure. This displacement is quantitatively captured through our holographic imaging system. Changes in the cell elasticity modulus and the morphology of the HUVEC, HCT 116, and C2C12 cells depending on the H_2_O_2_ concentration are shown in Fig. 1.

**Fig. 2:**
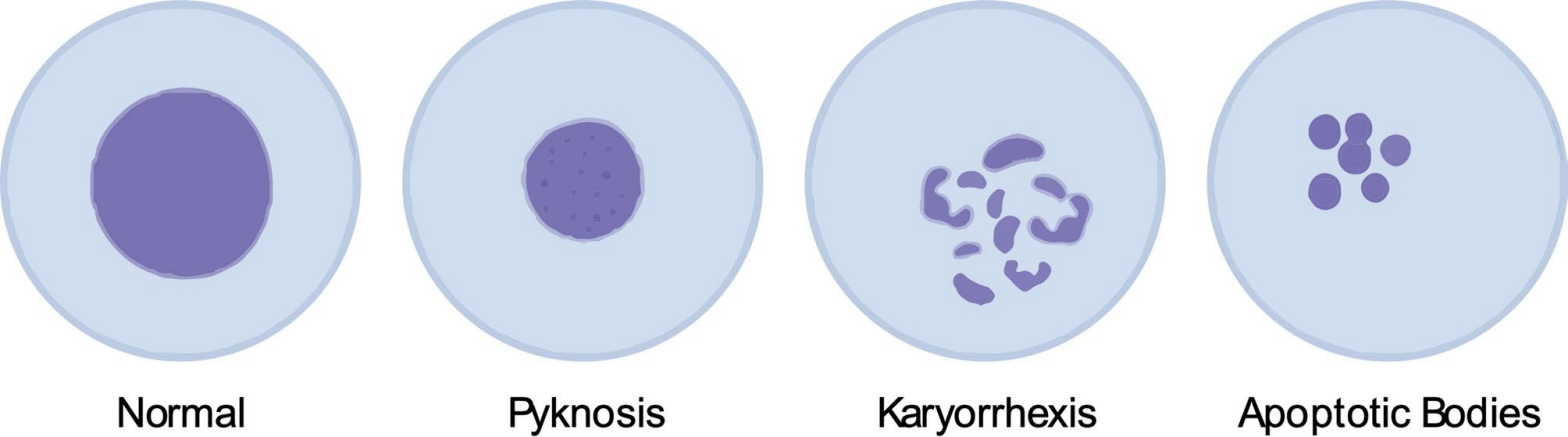
Nuclear morphology in apoptosis process.

Oxidative stress is one of the main results of cellular senescence and the susceptibility to apoptosis in the aging process varies according to different cell types^25^. When the apoptosis process begins, drastic changes in the morphology of the cell nucleus are observed. Chromatins are irreversibly condensed and the nucleus shrinks in the pyknosis state. Then, in the process of karyorrhexis, the pyknotic nucleus is fragmented and finally the cell becomes an apoptotic cell as shown in Fig. 2.

Results showed that in addition to the obvious morphological changes due to increasing H_2_O_2_ concentration, significant changes also occurred in the stiffness distribution of the cells. This may indicate apoptotic bodies that may contain micronuclei, chromatin remnants, and DNA fragments that disperse into the cytoplasm from the nucleus that is fragmented during apoptosis.

Results indicate that the mean elasticity modulus decreases due to the increasing H_2_O_2_ concentration for all cell lines. Figure 1a shows the morphological and mechanical changes that occur in HUVEC cells depending on the increased H_2_O_2_ concentration. Here, especially after the concentration of 1 mM H_2_O_2_, a significant shrinkage occurred in the cell nucleus, and at the concentration of 2 mM H_2_O_2_, the elasticity modulus in the nuclear region appears to have decreased. This situation can be characterized by degradation of DNAs and dissociation of the nuclear lamina in the apoptotic process due to oxidative stress.

While a similar situation was observed in HCT 116 cells, it was shown that this change was more pronounced at 2 mM H_2_O_2_ concentration as shown in Fig. 1b. At this point, the nucleus structure elasticity modulus at the nuclear region decreased and shrinkage began to be seen in cell morphology. This condition is also associated with nuclear fragmentation during the apoptosis process. In addition, the decrease in elasticity modulus due to increasing H_2_O_2_ concentration is associated with changes in the structure of the actin cytoskeleton and accordingly reorganization of the cytoskeletal structure. Although morphologically significant changes occurred in HCT 116 cells, when we compare the elasticity modulus changes in HUVECs and HCT 116 cells due to increasing H_2_O_2_ concentration, it has been shown that the elasticity modulus change is less in HCT 116 cells compared to its untreated control group. This may support the fact that cancer cells can ignore the signals that trigger them to enter the apoptosis process and do not undergo apoptosis when necessary.

Figure 1c shows that C2C12 cells started to shrink at 0.5 mM concentration of H_2_O_2_. The cells became more circular and softer at 1 mM concentration of H_2_O_2_. Then, karyorrhexis occurred at 1 mM concentration and stiffer elements in the nucleus spread into the cytoplasm, causing a softer elasticity modulus distribution in the center of the cell and a harder elasticity modulus distribution in the cytoplasmic regions.

**Fig. 3:**
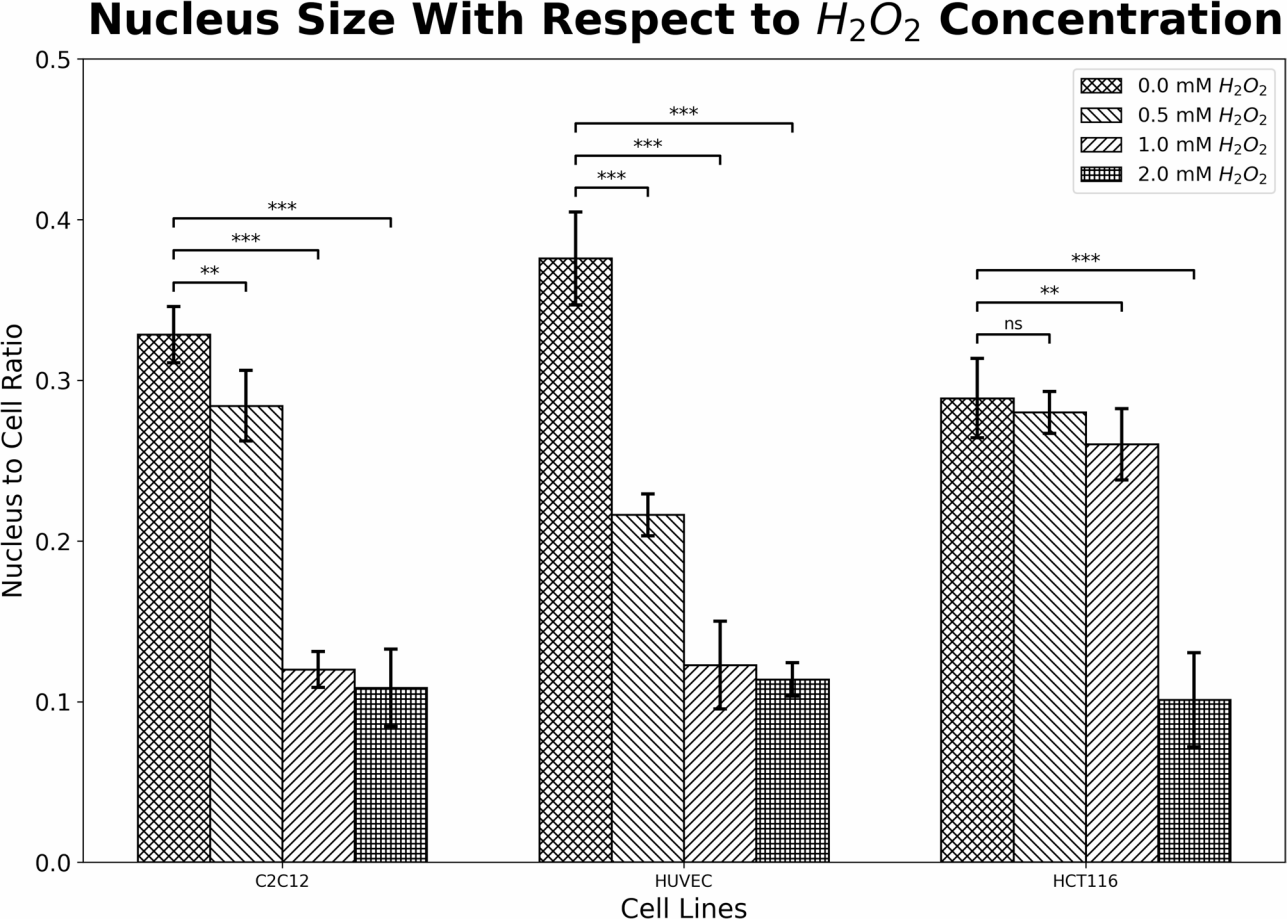
Nucleus to cell ratio for C2C12, HUVEC, and HCT 116 cell lines for 0, 0.5, 1, and 2.0 mM H_2_O_2_ concentrations. Results indicate that the nucleus to cell ratio decreases as H_2_O_2_ concentration increases. Statistical significance was tested using two-tailed t-test (*, **, and *** represent *p* < 0.05, *p* < 0.01, and *p* < 0.001 respectively).

Using the reconstructed thickness maps, we estimated the cell volume and nuclear volume for each cell. The cell boundaries and the nuclear boundaries were determined using a two-layered thresholding operation as described in the Methods section. The volumes were computed by summing the corresponding segment’s thickness over the pixel area​. The resulting nucleus-to-cell volume ratios are presented in Fig. 3 and show a decrease from 32.9% ± 1.75% (0 mM H₂O₂) to 10.9% ± 2.42% (2 mM H₂O₂) for C2C12 cells, from 37.6% ± 2.90% (0 mM H₂O₂) to 11.4% ± 1.04% (2 mM H₂O₂) for HUVEC cells, and from 28.9% ± 2.46% (0 mM H₂O₂) to 10.0% ± 2.93% (2 mM H₂O₂) for HCT116 cells, confirming nucleus shrinkage during apoptosis. The changes in nucleus size for all cell types with respect to cell volume were shown in Fig. 3. These results also support the nucleus shrinkage in apoptotic processes that change depending on the increased H_2_O_2_ concentration for all cell types.

**Fig. 4:**
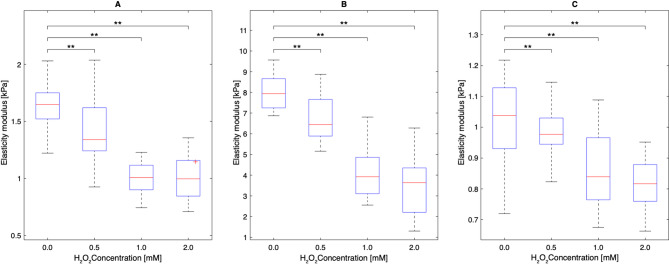
The mean elasticity modulus distribution of the (**A**) HUVEC, (**B**) C2C12, and (**C**) HCT 116 cells for 0 mM, 0.5 mM, 1 mM, and 2 mM concentrations of H_2_O_2_. Each group consisted of measurements taken from 40 single unfixed cells. Difference for each of the H_2_O_2_ concentrations was found to be statistically significant compared to the control group (** represents *p* < 0.01).

The mean elasticity modulus values of the cells that are exposed to H_2_O_2_ at different concentrations are given in Table 1 and also shown in Fig. 4 as box plots. Specific mean elasticity modulus values of the cells exposed to 0 mM (control group), 0.5 mM, 1mM, and 2 mM H_2_O_2_ were found to be 1.62 ± 0.38 kPa, 1.41 ± 0.35 kPa, 1.19 ± 0.44 kPa, and 1.11 ± 0.39 kPa, respectively for HUVEC cells. These values were measured as 7.61 ± 0.22 kPa, 6.88 ± 0.21 kPa, 4.71 ± 0.17 kPa, and 3.89 ± 0.20 kPa, respectively, for C2C12 cells. Finally, 1.08 ± 0.12 kPa, 0.99 ± 0.12 kPa, 0.88 ± 0.14 kPa, and 0.82 ± 0.13 kPa values were measured for HCT 116 cells. For each cell line, the H_2_O_2_ applied groups were found to be statistically different compared to the control group (*p* < 0.01). Each group consisted of measurements taken from 40 single unfixed cells. According to the AFM results of Sun et al. the elasticity modulus values of C2C12 cells were found as 3 kPa, 2 kPa, and 1.5 kPa for 0 mM, 0.5 mM, and 1 mM of H_2_O_2_ concentration, respectively (*p* < 0.05)^26^. AFM measurement could not be taken at 2 mM H_2_O_2_ concentration. It has been shown that the elasticity modulus results obtained with the acousto-holographic system were higher than the results taken by AFM. This can be explained by the different working principles of both methods, such as the fact that, unlike AFM, no probe is used in the acoustic holographic method and elasticity modulus is measured without any contact with the cell. On the other hand, in both methods, it was shown that elasticity modulus values decreased due to increasing H_2_O_2_ concentration. Furthermore, the mismatch in the results obtained from AFM and the acousto-holographic methods can also be attributed to unavoidable differences in the experimental conditions, which may affect the measurement outcomes.

**Table 1:** Specific mean elasticity modulus and standard deviation values of the HUVEC and C2C12 cells that are exposed to different H_2_O_2_ concentrations.

	HUVEC cells	C2C12 cells	HCT116
0 mM H_2_O_2_	Mean: 1.62 kPa, Std Dev: 0.38 Nucleus-cell ratio: 0.33	Mean: 7.61 kPa Std Dev: 0.22 Nucleus-cell ratio: 0.38	Mean: 1.08 kPa Std Dev: 0.12 Nucleus-cell ratio: 0.29
0.5 mM H_2_O_2_	Mean: 1.41 kPa, Std Dev: 0.35 Nucleus-cell ratio: 0.28	Mean: 6.88 kPa, Std Dev: 0.21 Nucleus-cell ratio: 0.22	Mean: 0.99 kPa Std Dev: 0.12 Nucleus-cell ratio: 0.28
1 mM H_2_O_2_	Mean: 1.19 kPa, Std Dev: 0.44 Nucleus-cell ratio: 0.12	Mean: 4.71 kPa, Std Dev: 0.17 Nucleus-cell ratio: 0.12	Mean: 0.88 kPa Std Dev: 0.14 Nucleus-cell ratio: 0.26
2 mM H_2_O_2_	Mean: 1.11 kPa, Std Dev: 0.39 Nucleus-cell ratio: 0.11	Mean: 3.89 kPa Std Dev: 0.20 Nucleus-cell ratio: 0.11	Mean: 0.82 kPa Std Dev: 0.13 Nucleus-cell ratio: 0.10

In addition, analysis of the control groups revealed that the cell elasticity modulus values followed the order: C2C12 > HUVEC > HCT 116. It has been shown that the decrease in elasticity modulus occurring in C2C12 cells due to increased oxidative stress is much more pronounced compared to other cell lines, and the least decrease was shown in HCT 116 cells, which is a cancer cell line. This indicates that cancer cells show higher resistance to apoptosis, while normal cells may be more significantly affected by oxidative stress and enter the apoptotic process faster.

In our study, elasticity modulus measurements were performed after 24 h of H₂O₂ incubation, at which stage apoptotic processes such as cytoskeletal depolymerization, nuclear shrinkage, and loss of elasticity modulus are dominant. Some previous studies have shown that short-term oxidative stress can induce actin polymerization and transient stress fiber formation, which may temporarily increase cell stiffness^27^. Therefore, it is reasonable to expect a biphasic mechanical response to H₂O₂: an initial transient stiffening due to stress fiber formation, followed by the progressive softening as observed at 24 h under sustained oxidative stress.

Our findings are in line with other acousto-optic approaches in the literature. For example, Frausto-Rea et al. used acoustic stimulation combined with holographic interferometry to measure stiffness changes in UV-irradiated skin tissue^28^​. They calculated stiffness by relating the known acoustic pressure (measured via sound levels) to the induced deformation, demonstrating a non-invasive means to assess biomechanical alterations. Similarly, our acousto-holographic system leverages the relationship between an applied acoustic force and the cell’s deformation to extract elasticity modulus values, confirming that holographic techniques are effective for biomechanical monitoring.

### Oxidative stress-induced cytoskeleton reorganization

Actin protein plays an important role in the regulation of apoptosis signaling. In fact, the actin skeleton functions both as a sensor and a mediator in the apoptosis process. To support the results obtained from the acousto-holographic system, changes in the actin cytoskeleton were monitored by a fluorescent microscope. The presence of F-actin under oxidative stress conditions depending on different H_2_O_2_ concentrations was detected by phalloidin staining.

**Fig. 5:**
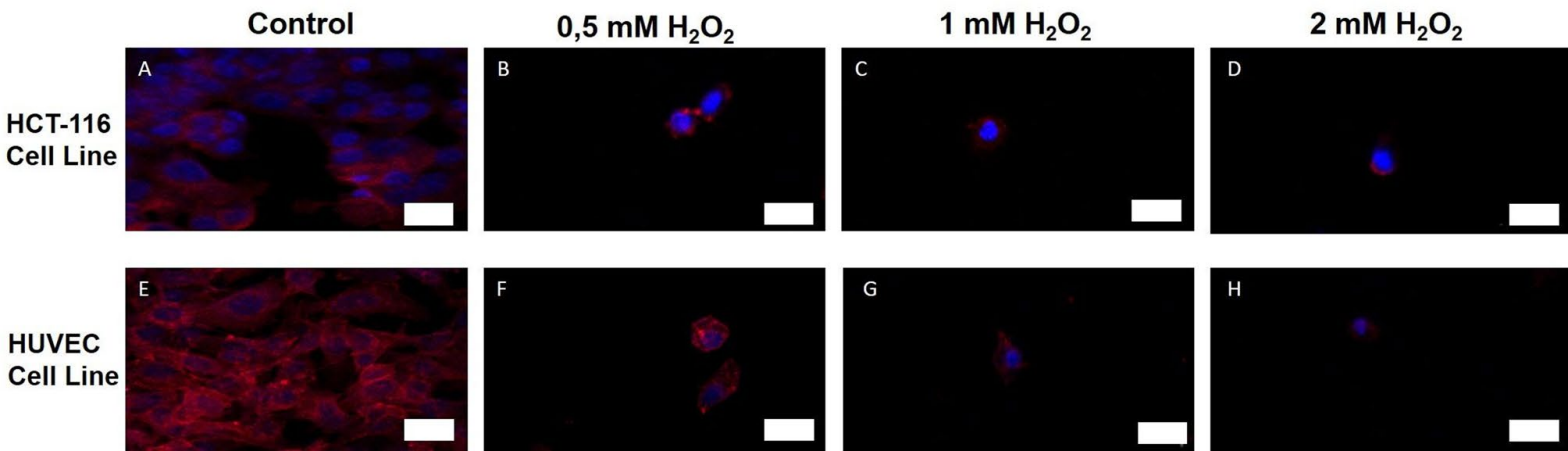
The microscopic images show the actin filaments of HUVEC and HCT 116 cells 24 h after H_2_O_2_ treatment. (**A**) HCT116 cell line control group (non-treated), (**B**) HCT 116 cell line treated with 0.5 mM H_2_O_2_, (**C**) HCT 116 cell line treated by 1 mM H_2_O_2_, (**D**) HCT 116 cell line treated by 2 mM H_2_O_2_, (**E**) HUVEC cell line control group (non-treated), (**F**) HUVEC cell line treated by 0.5 mM H_2_O_2_, (**G**) HUVEC cell line treated by 1 mM H_2_O_2_, (**H**) HUVEC cell line treated by 2 mM H_2_O_2_. Red color indicates actin filaments of the cells via phalloidin staining, blue color indicates cells’ nuclei staining by DAPI. White scale bar is 20 μm.

Actin is primarily responsible for the maintenance of cell stiffness. As seen in Fig. 5, phalloidin-dyed actin filaments decreased by increasing concentration of H_2_O_2_ for both cell lines. Especially after treatment of 1 mM and 2 mM H_2_O_2_, a significant reduction in the actin filaments was observed for both HUVEC and HCT 116 cells. This decrease in actin filaments due to the increasing H_2_O_2_ concentration on the cells was compared with the mean elasticity modulus values measured by acousto-holographic microscope. The results revealed that decreases in cell stiffness due to increased oxidative stress are associated with actin depolymerization and reconstruction of the cytoskeleton. Consequently, high levels of reactive oxygen species accumulate in the cells and cause changes in cell stiffness by damaging the cytoskeleton and cytoskeleton-cell membrane interaction. Oxidative stress has been shown to impair cytoskeletal integrity due to increased concentration and cause remodeling of the cytoskeleton.

### Morphological changes and apoptosis under oxidative stress

**Fig. 6:**
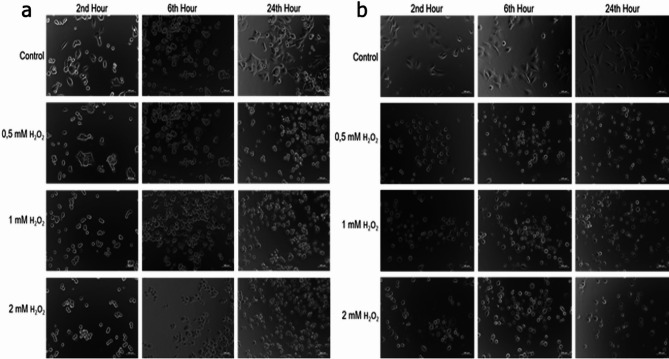
The microscopic images presented here show the morphology of the cells 2, 6, and 24 h after H_2_O_2_ treatment. (**a**) HCT 116 cell line, (**b**) HUVEC cell line. It can be seen that the morphological changes in cells exposed to H2O2 over time showed significant differences from the control group after two hours of treatment.

Morphological changes of the cells exposed to H_2_O_2_ were examined as a function of time and it was observed that the morphology of the cells treated with H_2_O_2_ for two hours started to change and characteristics of the cells were quite different compared to the control groups as seen in Fig. 6. Also, cells swelled and contained many heterogeneous vesicles in their cytoplasm. This situation may be attributed to cytoskeletal rearrangement and pH changes (6.7–8.5) in the culture conditions due to the oxidative stress. Moreover, apoptotic body formation began to be observed in the cells. Apoptosis studies were carried out after 24 h of H_2_O_2_ treatment.

**Fig. 7:**
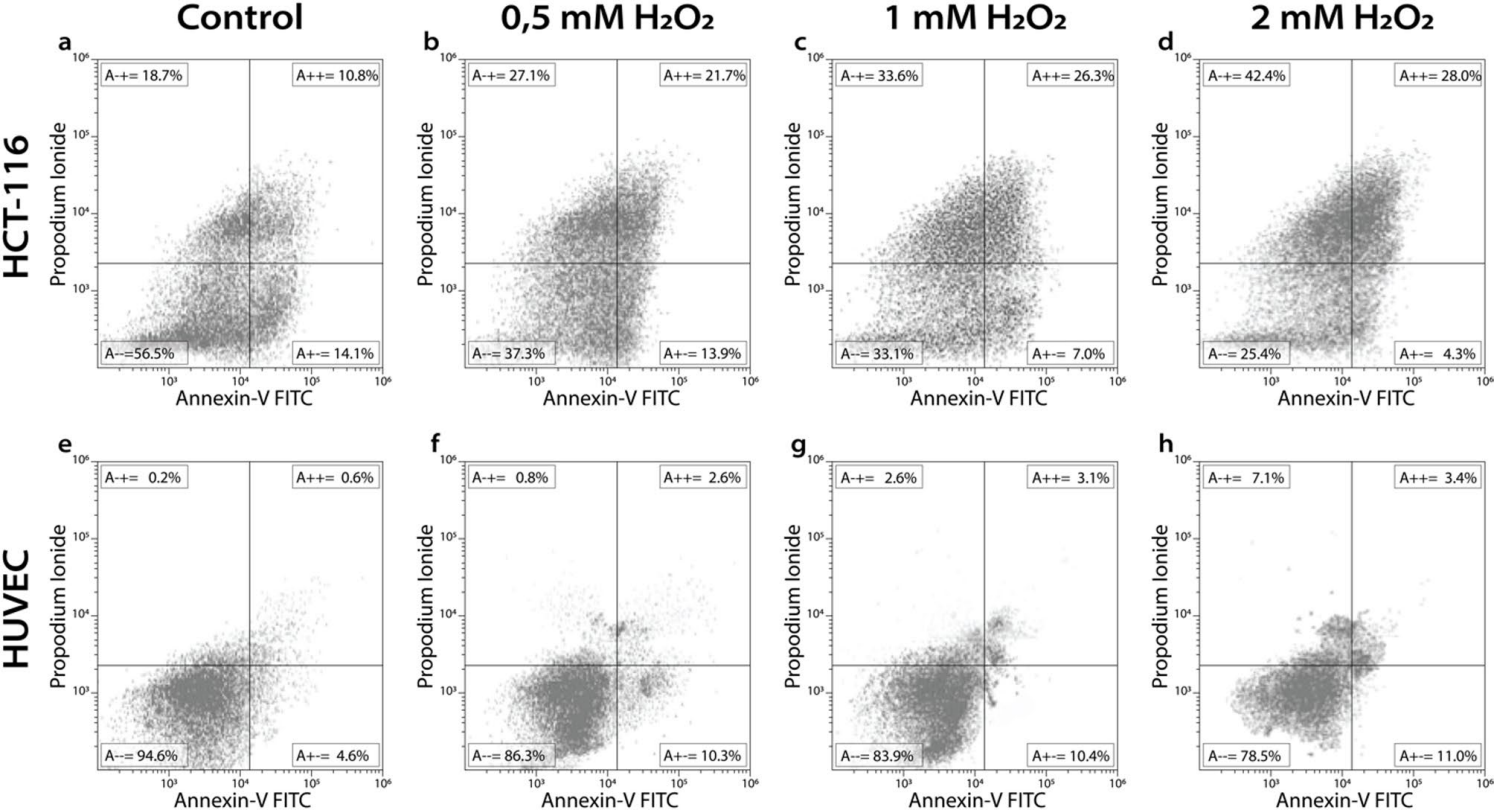
Annexin V-FITC/PI staining of HCT 116 and HUVEC cell lines (**a**) HCT 116 cell line control group, (**b**) HCT 116 cell line 0.5 mM H_2_O_2_, (**c**) HCT 116 cell line 1 mM H_2_O_2_, (**d**) HCT 116 cell line 2 mM H_2_O_2_, (**e**) HUVEC cell line control group, (**f**) HUVEC cell line 0.5 mM H_2_O_2_, (**g**) HUVEC cell line 1 mM H_2_O_2_, (**h**) HUVEC cell line 2 mM H_2_O_2_ treatment. The lower left quarter represents live cells, the lower right quarter represents early apoptotic cells, the upper right quarter represents late apoptotic cells, and the upper left quarter represents dead cells.

Oxidative stress in the cells plays a pivotal role in the development of apoptosis. According to the Annexin V-FITC/PI staining results, the number of apoptotic cells increases depending on the increasing H_2_O_2_ concentration as shown in Fig. 7. On the other hand, acousto-holographic analysis showed that nucleus structure of the HUVEC and HCT 116 cells exhibited a distinctly softer profile after treatment of 1 mM and 2 mM H_2_O_2_, and shrinkage has begun to be seen in cell morphology. These conditions were associated with nuclear fragmentation during the apoptosis process. Besides, cells undergo a characteristic morphological change during apoptosis in which the cytoskeleton is actively involved. In this process, the cytoskeleton is broken down by enzymes, and the cell shrinks, resulting in a more rounded shape which also supports acousto-holographic images. Late apoptosis is characterized by the stage of karyorrhexis, which begins with pyknosis and results in DNA fragmentation^29^. The cell became smaller and acquired a more rounded morphology. Late apoptotic cells at 0 mM, 0.5 mM, 1 mM, and 2 mM of H_2_O_2_ concentration were found as 10.8%, 21.7%, 26.3%, and 28%, respectively for HCT 116 cells. In HUVEC cells, these values were found to be 0.6%, 2.6%, 3.1%, and 3.4%. The results showed that the number of late apoptotic cells increased due to the increased H_2_O_2_ concentration in both cell lines.

Although powerful, the acousto-holographic platform has certain limitations. Spatial resolution is bounded (~ 1 μm lateral) by the optical system and acoustic wavelength, and throughput is limited by sequential reconstructions. Reproducibility requires precise calibration of acoustic and chamber conditions, and applying the method to complex 3D cultures or tissues may be challenged by scattering and acoustic attenuation.

## Conclusion

In this study, we propose a platform that allows us to analyze the morphology of the cell under oxidative stress in a high-resolution, label-free manner, while also mapping the elasticity modulus of cells. The results showed that the cells have a softer characteristic due to the increasing H_2_O_2_ concentration and this has been associated with cytoskeletal rearrangement depending on apoptosis, supported by performing comparative analyzes. To sum up, this method offers a non-invasive and label-free approach that enables the real-time examination of changes in the morphological and mechanical properties of live cells. Our findings are consistent with prior studies that measured the mechanical response of cells to external agents^30^. Moreover, through this methodology which offers both qualitative and quantitative analysis, it may be possible to test the effectiveness of the anticancer and antioxidant drugs, also to examine cell mechanics in a large cell population at the single cell level, and to identify cells with different characteristics in the population. Finally, when compared to equivalent techniques, this method offers an effective and high-speed analysis at the single cell level.

## Materials and methods

### Materials

#### PDMS fluidic chamber

Polydimethylsiloxane (PDMS) polymer, and Sylgard 184 elastomer curing agent were obtained from Dow Corning, Midland, MI. Microscope cover glass slides (0.13–0.17 mm thick), and 25 × 7.1 × 0.50 mm Piezo Bimorph Actuators (PZT, SM411) were purchased from Thermo Fisher Scientific, Inc., and Steiner & Martins, Inc., respectively.

#### Cell culture

Human umbilical vein endothelial cells (HUVEC), mouse myoblast cells (C2C12), and human colorectal carcinoma cells (HCT 116) were purchased from American Type Culture Collection-ATCC (Manassas, VA, USA). DMEM/F12 (Dulbecco’s Modified Eagle Medium/Nutrient Mixture F-12) medium, McCoy’s 5 A medium, Fetal Bovine Serum (FBS), Trypsin-EDTA, and penicillin–streptomycin were purchased from Gibco, and Phosphate Buffered Saline (PBS) was supplied from Sigma-Aldrich.

#### Optical equipment

The holographic imaging setup was based on a phase shifting inline Mach-Zehnder interferometer. As a coherent light source, we used a 527 nm, 10 mW He-Ne laser. A high-frequency piezo actuator (New Focus, Picomotor 8302) actuated in 1000 steps per second was used for phase-stepping. An algorithm for real-time holographic reconstruction using a continuously shifting piezo actuator and a high-speed CMOS camera (ZEISS, AxioCam 702 mono) was developed. The CMOS camera was synchronized to the motion of the piezo actuator and was programmed to capture an image at each step. For each frame we predicted the phase difference between the last two consecutive frames and used the last period of the captured interferograms for holographic reconstruction.

### Methods

#### Manufacturing of the PDMS fluidic chamber

**Fig. 8:**
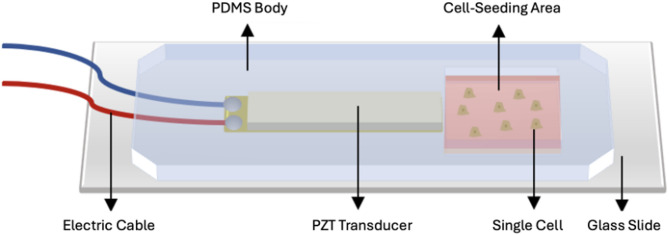
3D illustration of the PZT transducer-embedded fluidic chamber. The PDMS fluidic chamber was produced by a soft lithography technique. Cells were seeded in the fluidic chamber with a concentration of 5 × 10³ cells, incubated, exposed to H_2_O_2_ for 24 h with various conditions, and were subjected to acoustic stimulation via the PZT transducer in order to measure the elasticity modulus distribution.

The PDMS fluidic chamber was produced by a soft lithography technique. Firstly, pre-polymer and curing agent were mixed with a 10:1 (w/w) ratio. The mixture was degassed for 30 min in a desiccator to remove any air bubbles from the polymer. Then, the polymer was poured into a mold and a PZT transducer was placed on the polymer. The final structure was baked at 60 °C for 3 h and a partition with the dimensions of 12 mm x 12 mm x 5 mm was cut and then peeled off from the mold. Irreversible bonding between PDMS structure and glass slide was provided by oxygen plasma application at 400 mTorr pressure and high power for two minutes. A 3D drawing of the manufactured fluidic chamber is shown in Fig. 8.

#### Cell culture and H_2_O_2_ treatment

HUVEC, C2C12, and HCT116 cells were used for cell culture experiments. HUVEC and C2C12 cell lines were cultured in DMEM/F-12 medium and HCT116 cells were cultured in McCoy’s 5 A medium supplemented with 10% fetal bovine serum and 0.5% penicillin–streptomycin. Cells were incubated at 37 °C in 5% CO_2_. The confluent culture was trypsinized and centrifuged at 1,000 rpm for 5 min. The supernatant was discarded and the cell number in the pellet was counted with a hemocytometer. Afterwards, cells were seeded in the fluidic chamber with a concentration of 5 × 10³ cells, mL^− 1^ and incubated at the standard cell culture conditions before the H_2_O_2_ experiment. Finally, cells were exposed to H_2_O_2_ for 24 h with the concentrations of 0.5 mM, 1 mM, and 2 mM, respectively, and mechanical and morphological changes under oxidative stress conditions were examined periodically via the acousto-holographic microscope.

#### Holographic vibration measurement and determination of cell elasticity modulus

In order to measure the mechanical and morphological changes induced on the cells by acoustic force produced by the PZT transducer, we use an interferometric imaging system that is based on the phase shifting Mach-Zehnder setup. At each step of the piezo motors, the CMOS camera captures an image with the following form:

1 $$\:{I}_{ij}^{t}={A}_{ij}+{B}_{ij}cos({\varphi\:}_{j}+{\delta\:}_{i})$$

Where $\:t$ denotes the theoretical value, $\:i$ represents the $\:i$th phase-shifted interferogram ($\:i$ =1,2,…,M), and $\:j$ denotes the individual pixel locations in each image ($\:j$ =1,2,…,N). Here $\:{A}_{ij}$ is the background intensity, $\:{B}_{ij}$ is the modulation amplitude, $\:{\varphi\:}_{j}$ is the angular phase information, and $\:{\delta\:}_{i}$ is the phase-shift amount of each frame. Combining the information obtained from multiple interferograms captured throughout a period of the vibration, the displacement amplitude can be extracted. Further details of the methodology were given in a previous study^23^.

#### Reconstruction of elasticity modulus maps

We collect 50 interferograms at each of 20 phase steps corresponding to a phase shift of π/10 and employ a wavelet transform-based phase matching algorithm to align consecutive interferograms for each phase step. After alignment, a least-squares minimization technique minimizes phase difference errors, enabling the construction of a continuous video showing the cell surface’s vibration pattern. The interferograms are then sorted into 50 bins, each representing 1/50th of a vibration period, and filtered to reduce noise. Phase unwrapping is performed using the preconditioned conjugate gradient (PCG) method^31^, which involves computing the discrete cosine transform of the interferogram, solving the discrete Poisson equation, and applying the inverse cosine transform. Following phase reconstruction and thickness extraction, we generate a thickness map and iteratively determine the elasticity modulus coefficient using a two-dimensional linear-elastic membrane model to best match the experimental data. A comprehensive description of the elasticity modulus calculation is given in the next section. Each elasticity modulus map was reconstructed using interferograms acquired at a resolution 1920 × 1216 in ~ 10 s, corresponding to a sampling rate of ~ 4.28 MS/s (Mega samples per second). The lateral resolution of the elasticity modulus distribution calculation is dependent on the lateral resolution of the holographic imaging system itself.

#### Elasticity modulus calculation

Throughout an acoustic field, a non-zero time-averaged stress is created by non-linear terms in the Navier-Stokes equations. This stress is discontinuous across the boundary of a cell and its effect can be approximated in the non-viscous regime as a stress difference^1^:

2 $$\:\varDelta\:\varPi\:={\varPi\:}_{i}-{\varPi\:}_{o}$$

where $\:{\varPi\:}_{i}$ and $\:{\varPi\:}_{o}$ are the j on the inside and the outside of the membrane, respectively, given to second order as^2^

3 $$\:{\varPi\:}_{jk}=-\langle\:P-{P}_{o}\rangle\:{\delta\:}_{jk}-{\rho\:}_{0}\langle\:{\mathrm{v}}_{j}{\mathrm{v}}_{k}\rangle\:$$

where $\:{\delta\:}_{jk}$ is the Kronecker delta, $\:\langle\:\dots\:\rangle\:$ represents the time average. The second term on the right-hand side is the Reynolds stress, representing the time-averaged transport of the momentum density $\:{\rho\:}_{0}{v}_{j}$ with velocity $\:{v}_{k}$ in the direction of the surface normal. The time averaged mean Eulerian excess pressure is given by^3^

4 $$\:\langle\:P-{P}_{o}\rangle\:=\frac{1}{2{\rho\:}_{0}{c}_{0}^{2}}\langle\:{p}^{2}\rangle\:-\frac{{\rho\:}_{0}}{2}\langle\:{v}^{2}\rangle\:$$

with $\:{\rho\:}_{0}$, $\:{c}_{0}$, $\:p$, and $\:v$ denoting the quiescent fluid density, ambient speed of sound, first order acoustic pressure, and first order acoustic velocity, respectively.

In our calculations, the quiescent fluid density ($\:{\rho\:}_{0}$) was taken as 1000 kg/m³ for the cell culture medium at 37 °C^32^. This value is a property of the fluid and does not depend on the elasticity modulus of the cell. The ambient speed of sound ($\:{c}_{0}$) was set to 1510 m/s, which is the standard value for aqueous biological media^33^. This constant is determined by the medium’s compressibility and density, again independent of the cell’s elasticity modulus. The first order acoustic pressure ($\:p$) was determined to be approximately 70 Pa based on COMSOL simulations (as detailed in the next sections). $\:p$ is a controlled input from the transducer and is not influenced by the cell’s elasticity modulus. The first order acoustic velocity ($\:\mathrm{v}$) refers to the particle velocity in the medium, not within the cell, and was calculated from the acoustic pressure using the relationship $\:\mathrm{v}=p/\left({\rho\:}_{0}{c}_{0}\right)$. These values are properties of the surrounding medium rather than the cell itself, and therefore do not depend on cell elasticity modulus.

Through the formulation given in (4), longitudinal modulus ($\:{E}_{L}$) of the membrane is calculated through the linear relation:

5 $$E_{L} = \Pi _{{jk}} /\varepsilon$$

where ε is the strain. We assume predominantly axial deformation due to the acoustic wave; thus, the calculated elasticity modulus corresponds to the longitudinal modulus of the cell, where lateral deformation is constrained. In our acousto-holographic system, the strain $\varepsilon$ is calculated from the measured displacement $\:d$ using

6 $$\varepsilon = d/\phi _{0}$$

where $\:d=\varphi\:-{\varphi\:}_{0}$ with $\:{\varphi\:}_{0}$ denoting the initial thickness of the sample and $\:\varphi\:$ corresponding to the thickness at the instant of the acoustic pressure. Employing an elastic model to interpret cellular responses under acoustic stress introduces certain limitations due to the inherent viscoelastic nature of biological cells. While the elastic model simplifies the complex interactions within cellular structures by assuming an instantaneous and reversible response to mechanical stress, it overlooks the time-dependent and irreversible behaviors typical of viscoelastic materials.

#### Nuclear volume and elasticity modulus determination

The nucleus was identified in our holographic images based on optical density differences between the nucleus and cytoplasm. Cell boundaries were detected using an intensity-based thresholding algorithm, while nuclear boundaries were determined using a secondary thresholding operation calibrated to the higher optical density of the nucleus.

Cell volume was calculated by integrating the reconstructed thickness over the entire cell area. Similarly, nuclear volume was calculated by integrating the thickness over the identified nuclear region. The nucleus-to-cell ratio reported in our results represents the ratio of these calculated volumes. We acknowledge that this approximation neglects a thin layer of cytoplasm that may be present above and below the nucleus. However, because adherent cells typically flatten when attached to a substrate, these cytoplasmic regions are minimal, making our approximation a reasonable estimate of the actual nuclear volume.

Nuclear elasticity modulus was determined by isolating the elasticity modulus values within the identified nuclear region of the map. When we state that “the nucleus became softer,” we are referring to a decrease in the mean elasticity modulus value within this nuclear region. This approach allows us to monitor changes in nuclear mechanical properties independently from the cytoplasmic regions of the cell.

#### Calculation of the acoustic pressure constant

To determine the acoustic pressure constant used in our elasticity modulus calculations, finite element analysis (FEA) was performed using COMSOL Multiphysics^®^. In this simulation, the acoustic pressure field was modeled by coupling the “Pressure Acoustics” module with the “Solid Mechanics” module through the “Acoustic-Structure Interaction” interface. The acoustic signal was defined as a spherical wave radiation source positioned at the bottom-center of one of the side walls, representing the output of the PZT transducer. A transient analysis was carried out over the interval from 0 to 1 × 10⁻³ s with a step size of 1 × 10⁻⁵ s to capture the evolution of the pressure field. A subsequent frequency analysis was then performed to resolve phase variations and confirm the steady-state pressure distribution. The resulting simulation provided a quantitative estimate of the first-order acoustic pressure at the cell location, which was adopted as the acoustic pressure constant in our experimental calculations.

#### Fluorescence imaging of actin filaments

The presence of F-actin was detected by phalloidin staining (Alexa Fluor™ 647 Phalloidin, Life Tech, Waltham, USA). HUVEC and HCT116 cell samples were washed once with PBS and fixed with ice-cold 3.7% paraformaldehyde in PBS for 10 min and the samples were washed with PBS twice. Samples were then permeabilized with 0.1% Triton X-100 in PBS for 3 to 5 min and stained with phalloidin. Samples were imaged using a Zeiss LSM 800 confocal laser scanning microscope (Carl Zeiss, Inc.). All images were analyzed using the LSM Software Zen 2 (Blue Edition).

#### Assessment of apoptosis

Apoptosis was determined by staining cells with Annexin V-fluorescein isothiocyanate (FITC)/propidium iodide (PI) apoptosis kit (BD Biosciences, Franklin Lakes, NJ, USA). HUVEC and HCT 116 cells (1 × 10^6^ cells/mL) were resuspended in a binding buffer containing Annexin V-FITC and PI. 5 µl of Annexin V-FITC was then added to these cells and analysis was performed with a FACStar flow cytometer (Becton-Dickinson, Franklin Lakes, NJ, USA).

### Statistical analysis

Mean elasticity modulus and nucleus-to-cell ratio values for H2O2-treated groups were compared with the control using two-tailed t-tests as shown in Figs. 3 and 4. Statistical significance levels are indicated by *, **, and *** corresponding to *p* < 0.05, *p* < 0.01, and *p* < 0.001, respectively.

## Data Availability

The datasets generated during and/or analysed during the current study are available from the corresponding author on reasonable request.
